# Research on the impact of graphene oxide in feed on growth and health parameters in calves

**DOI:** 10.3389/ftox.2025.1560078

**Published:** 2025-07-30

**Authors:** Tereza Aulichova, Sylvie Skalickova, Kopec Tomas, Pompido Chilala, Pavel Horky

**Affiliations:** ^1^ Department of Animal Nutrition and Forage Production, Mendel University in Brno, Brno, Czechia; ^2^ Department of Animal Breeding, Faculty of AgriSciences, Mendel University in Brno, Brno, Czechia

**Keywords:** graphene oxide, adsorbent, animal, toxic, cattle

## Abstract

Mycotoxins, as feed contaminants, pose serious health risks and cause significant economic losses on farms. The selection of an appropriate and effective adsorbent remains a key challenge for many researchers. Graphene oxide (GO) and its derivatives have garnered interest due to their exceptional physicochemical properties. However, the increasing use of GO necessitates a thorough investigation into its potential toxic impacts on animal and human health, as well as the environment. This study evaluates the effects of GO as a feed additive on calf health. Ten calves (100 ± 6 kg) participated in a 20-day experiment: five in the control group (C) and five in the experimental group (T). The control group (C) received feed without GO, while the experimental group (T) was fed a diet containing 30 g of GO/kg/day. Key parameters evaluated included growth performance, biochemical markers (ALT, AST, ALP), and mineral levels (Ca, P, Mg, K, Na, Cl, Fe, Cu, Zn). The average weight gain was 16.20 ± 0.32 kg in the control group and 15.40 ± 0.26 kg in the GO group, with no statistically significant difference (p > 0.05). Calves fed GO-enriched feed exhibited significant reductions in Fe (p = 0.041) and Zn (p = 0.0006) levels, while Mg increased significantly in the control group (p = 0.029). Liver parameters in group T showed significant increases in ALT (p = 0.022), AST (p = 0.027), and ALP (p = 0.015) after 20 days. Additionally, GPx activity was significantly decreased in the GO group (p = 0.011). These results suggest that GO at a dose of 30 g/kg/day in feed can negatively affect calf health.

## 1 Introduction

Contamination of food and agri-food products with mycotoxins is a serious global problem that poses significant safety risks to livestock, humans, and the economy (Khodaei et al., 2020). Low molecular weight mycotoxins (Tolosa et al., 2021) are products of the secondary metabolism of toxigenic fungal species (Kemboi et al., 2020; Jaynes et al., 2007; Penagos-Tabares et al., 2023), including *Alternaria* spp., *Aspergillus* spp., *Fusarium* spp., and *Penicillium* spp (Santos et al., 2022). The greatest health risks to livestock and humans are posed by aflatoxins (AF), ochratoxin A (OTA), deoxynivalenol (DON), T-2/HT-2 toxin, fumonisins (FUM) and zearalenone (ZEN) toxins (Khodaei et al., 2020). Emerging mycotoxins include beauvericin (BEA) and enniatins (ENN) (Tolosa et al., 2021). Ruminants are reported to be less sensitive than monogastric animals, which is attributed to microbial activity in the rumen that can modify the chemical structure of mycotoxins into less toxic compounds. (Kemboi et al., 2020). However, some studies report that many rumen bacteria can be inhibited by mycotoxins (Jaynes et al., 2007). The rumen microbiome can metabolize mycotoxins, converting ochratoxin A (OTA) into less toxic compounds like ochratoxin-α and phenylalanine, but it can also produce more toxic substances, such as α-zearalenol, compared to the original mycotoxin (Penagos-Tabares et al., 2023). Due to their global prevalence, it is nearly impossible to avoid the presence of mycotoxins in the food chain; however, their levels can be controlled through proper agricultural practices and decontamination procedures (Santos et al., 2022). Although the primary objective of the agriculture and the feed industries is to prevent mycotoxin contamination in the field and during storage, the complete absence of fungi and mycotoxins in livestock rations cannot be guaranteed (Vila-Donat et al., 2018). Based on these factors, the EU approved the use of mycotoxin detoxifying agents by including a new functional group within the category of technological additives, defined as “*substances that may inhibit or reduce absorption, promote the excretion of mycotoxins or modify their mode of action*”, as indicated in Regulation (EC) No 1831/2003 (Commission, 2009). These additives can bind mycotoxins to their surface (adsorbents) or degrade or convert them into less toxic metabolites (biotransformation) (Vila-Donat et al., 2018).

Carbon is one of the most abundant elements on our planet, occurring naturally in many allotropic forms, such as diamond, graphene, fullerene, *etc.* Carbon nanomaterials (CNMs) consist of sp^2^-bonded graphitic carbon and are characterized by high chemical resistance, excellent mechanical properties, and very low weight (Simon et al., 2019; Hassanpour et al., 2021). Graphene is considered the simplest form of carbon, the thinnest (Priyadarsini et al., 2018), lightest, and strongest material. Graphene is not widely used for biological applications due to its high hydrophobicity (Ghulam et al., 2022). Graphene oxide (GO) in oxidized form is preferable for biological purposes because of being hydrophilic (Ou et al., 2016; Ng and Shamsi, 2022; Özsobaci and Ergün, 2023; Anand et al., 2019). GO is a chemically modified form of graphene (Zhao et al., 2022) which has a simple mononuclear 2D structure of carbon atoms bonded by sp^2^ and sp^3^ bonds (Kashif et al., 2022), classifying it as a two-dimensional material. GO has many oxygen-containing functional groups on its surface, including hydroxyl, carbonyl, carboxyl, epoxy, lactone, and phenolic groups (Zhao et al., 2022; Lu et al., 2018; Itoo et al., 2022; Tanveer et al., 2020). GO also exhibits high biocompatibility and potential for surface functionalization with great potential in various fields (Liu et al., 2021), including drug delivery (Itoo et al., 2022; Zhao et al., 2023; Shareena et al., 2018), biosensors (Shareena et al., 2018), environmental remediation (Zhao et al., 2023), water filtration (Ajala et al., 2022), tissue engineering, and optical imaging (Ghazimoradi et al., 2022).

Due to its structure, GO can bind mycotoxins with different chemical structures, such as those containing an aromatic ring or an aliphatic chain. Selective adsorption of these aromatic compounds occurs via strong pi–pi (π–π) interactions or hydrogen bonding (Martin-Folgar et al., 2022; Bai et al., 2018; Gao et al., 2012; Abbasi Pirouz et al., 2021). Other possible interactions for carbon nanoparticles include the hydrophobic effect, as well as covalent and electrostatic interactions (Yang and Xing, 2010; Creighton et al., 2013). Adsorption on carbon is preferred for molecules with low solubility, partial hydrophobicity, positive charge, and those with conjugated π-bonds, which impart polarity and allow π-π interactions with GO (Sanchez et al., 2012). The binding of these elements to GO is mediated by electrostatic interactions, with the strength of the interaction being determined by the polarity of the reaction medium and the magnitude of the charges (Magne et al., 2022; Horky et al., 2020).

Scientists are faced with the challenge of balancing the positive therapeutic effects with the potential negative side effects. Several *in vivo* studies have shown that graphene nanoparticles accumulate in living organisms, posing potential health risks to animals (Szmidt et al., 2016; Ema et al., 2016; Dasmahapatra et al., 2019; Li et al., 2016; Bantun et al., 2022). It is important to note that GO has some disadvantages, such as its great diversity due to the presence of various oxygen-containing groups. The representation of individual oxygen groups can vary depending on the preparation method, which may adversely affect the desired properties (Sedajová et al., 2023). For instance, studies have shown that the temperature and water content during graphite oxidation influence the types and amounts of oxygen functional groups in GO, which subsequently affect its electrical conductivity and structural integrity (Chen et al., 2019). By altering the reaction temperature during GO synthesis, the type and content of individual oxygen functional groups can be controlled. At 50°C, GO samples were rich in hydroxyl and carboxyl groups, resulting in improved hydrophilicity and higher moisture content. Conversely, at 100°C, epoxide groups formed with a reduction in overall oxygen content, leading to decreased hydrophilicity and lower moisture retention (Luo et al., 2018). Another study highlights that the water content in the reaction medium affects the distribution of functional groups, interlayer spacing, and stability of the GO suspension. GO prepared with lower water content contained more hydroxyl and epoxide groups, whereas higher water content led to increased amounts of carbonyl groups (Zhang et al., 2021). Changes in the structure of GO have a direct impact on its interaction with biological systems. Studies indicate that GO with a higher content of oxygen groups, especially hydroxyl and epoxide groups, exhibits lower cytotoxicity and better biocompatibility. In contrast, reduced GO (rGO) with lower oxygen content may cause greater DNA damage and higher production of reactive oxygen species (ROS) (Ou et al., 2021). Another disadvantage may be non-specific adsorption, which could result in the adsorption of essential nutrients (Bytešníková et al., 2023; Long et al., 2012). Some authors suggest that proper functionalization can reduce toxicity risks (Ahmadi et al., 2017). For example, research by Wang et al. (2011) found that the lower the positive surface charge of GO, the milder its toxic effect on cells (Wang et al., 2011).

Recent research shows that graphene oxide (GO) is finding increasing applications across a variety of fields, including environmental technologies and water filtration (Zubair et al., 2024) (Anegbe et al., 2024) (Shah et al., 2023) (Tariq et al., 2022), optoelectronics and photonics (Wu et al., 2023), supercapacitors and electrodes for energy storage (Chen et al., 2022) (Biru et al., 2022) (Yim et al., 2021), biomedicine and neurosurgery (Chen et al., 2022) (Biru et al., 2022) (Yim et al., 2021), as well as agriculture (Tariq et al., 2022) (Sharma et al., 2025), for example, in the controlled release of macronutrients into soil (Zhang et al., 2025). Its use in the food industry is also growing, particularly as a component of antibacterial packaging materials (Sharma et al., 2025) and for the detection of contaminants and foodborne pathogens (Yadav et al., 2025).

To date, no experiments have been conducted to investigate the feeding of GO to livestock. In our study, we focused on the effects of feeding GO to calves. The main aim of this experiment was to test the hypothesis that GO can negatively affect the performance and biochemical parameters of calves. The increasing application of GO necessitates the study of its toxicity in organisms and the environment, as well as its interaction with macro- and micronutrients, specifically minerals, in organisms. In this study, we focused on the effect of GO on the growth performance of calves, its impact on health parameters, and its interaction with minerals in calves.

## 2 Materials and methods

### 2.1 Chemicals

GO (size: 300–800 nm, thickness: 0.7–1.2 nm) was purchased from Cheptubes Inc. (Cambridgeport, United States).

### 2.2 Animal experiment

All animal experiments were conducted in compliance with European regulations (EU legislation). The experiment included 10 calves, six bulls and four heifers of the Chester breed, all of the same age (between 3 and 4 months) with an average weight of 100 kg. Due to technical limitations, the calves were weighed as a group using a large-scale platform, and the mean weight per calf was calculated. This approach did not allow for capturing individual variations in feed intake or weight gain. The experiment took place on the farm of ALA, a.s. in Řepníky (Czech Republic). The calves were individually ear-tagged and housed in a common pen with straw bedding throughout the entire observation period. The animals were divided into two groups: the control group, consisting of five animals (three bulls and two heifers), and the experimental group, consisting of five animals (three bulls and two heifers). Although the number of animals was limited, the study was designed as a pilot trial to evaluate the initial biological effects of GO in calves, following a similar approach to other studies involving feed additives in ruminants (Szacawa et al., 2021). The feeding experiment lasted for 20 days. GO was diluted with flour in a ratio of 30% graphene oxide and 70% flour, then homogenized in the feed. Each calf, weighing approximately 100 kg, was fed a mixed ration containing 5 kg of dry matter (DM) per day, with 30 g of graphene oxide (GO) administered per animal per day, divided into two equal doses of 15 g GO/head given twice daily. The selected GO dose corresponds to approximately 30 g/kg body weight per day and was based on previously published *in vivo* and *in vitro* studies, which demonstrated biological effects of GO without significant toxicity at comparable or lower dosages in rodent models (Horky et al., 2020; Shen et al., 2022; Liang et al., 2015). Calves were fed twice a day, and any feed refusals were collected, weighed, and recorded after each feeding to accurately determine individual feed intake. Calves were also weighed regularly to monitor their growth.

Prior to the start of the 20-day experimental period, the GO feed mixture was introduced to the experimental group for 3 days as a habituation phase to allow the calves to get used to the taste and the black colour of the preparation. These 3 days were not included in the experimental exposure period.

### 2.3 Feed ration analysis

The feed ration was formulated and analysed using AgroKonzulta (Žamberk, Czech Republic). It consisted of meadow hay, corn silage, alfalfa silage with 50% pea, and M/premium start non-GMO. The composition and nutritional values of the feed ration are shown in Tables 1 and 2. The evaluation of feed quality and nutrient composition followed standard procedures consistent with those described by Horký et al. (2014) and Horky et al. (2017).

**TABLE 1:** Ingredient composition of the calf diet.

Ingredient	Piece/Day/Kg
Meadow hay	0.300
Corn silage	1.400
Alfalfa silage 50% + peas	1.004
M/Premiumstart non-GMO	2.200
Total	4.904

**TABLE 2:** Chemical composition of the feed rations (dry matter).

Indicator	Composition	Norm-min	Norm-max	Fulfillment (%)
Dry matter (g)	3,188.3	2,840.4	3,787.2	100.00
N-substances (g)	556.60	493.05	640.96	100.00
PDIN (g)	355.45	337.63	371.39	100.00
PDIE (g)	325.17	337.63	347.76	96.31
Fiber (g)	469.27	263.40	526.80	100.00
NEL (MJ)	21.253	20.634	21.253	100.00
Ca (g)	27.949	21.500	37.625	100.00
P (g)	15.835	14.100	23.970	100.00
Na (g)	6.403	3.780	9.450	100.00
K (g)	37.67	10.20	40.80	100.00
Mg (g)	8.817	5.810	12.450	100.00
Fe (mg)	344.863	240.350	2,783.000	100.00
Cu (mg)	69.56	29.83	219.80	100.00
Mn (mg)	443.39	149.91	1,104.60	100.00
Zn (mg)	397.25	149.91	1,104.60	100.00
Se (mg)	1.811	0.903	3.800	100.00
I (mg)	4.897	1.197	7.560	100.00
Vitamin A (IU)	44,000	41,028	189,360	100.00
Vitamin D (IU)	4,400	4,102	18,930	100.00
Vitamin E (mg)	127.47	66.29	284.10	100.00
Lactic acid (g)	48.41			
Acetic acid (g)	8.57			
Butyric acid (g)	0.24			
DIN/PDIE	1.093	1.000	1.160	100.00
NEL/Dry matter	6.666	6.308	8.029	100.00
%NL/Dry matter	17.458	16.445	24.668	100.00
Fiber/Dry matter	14.718	9.737	16.692	100.00
Ca/P	1.765	1.449	2.287	100.00
K/Na	5.884	1.889	9.445	100.00
% starch in dry matter	28.943			100.00

### 2.4 Sampling and analysis of feed samples for mycotoxins

Samples of feed (Corn silage, alfalfa-pea silage and starter) were analysed by ELISA in the laboratory of SVU Jihlava (State Veterinary Institute Jihlava) (Table 3).

**TABLE 3:** Mycotoxin content in kg of dry matter of the daily feed ration (mg/kg).

Ingredient	Pcs/Day/Kg	DON	ZEN
Meadow hay	0.300	<0.10	<0.05
Corn silage	1.400	0.23	<0.05
Alfalfa silage 50% + peas	1.004	0.61	0.07
M/Premiumstart	2.200	<0.10	<0.05
Total	4.904	0.84	0.7

### 2.5 Blood sample collection and analysis

Blood samples were collected from the vena jugularis of the calves by a veterinarian for analysis of blood and liver parameters on day 0 (prior to any exposure to the GO mixture, including the habituation phase) and on day 20 (the last day of the experimental period). The samples were frozen immediately after collection and subsequently analysed. The following blood parameters were analysed: albumin, Ca, P, Mg, K, Na, Cl, Fe, Cu, Zn, ALT, AST, and ALP.

The determination of albumin was performed in whole heparinized blood serum using the IDEXX VetTest analyser. The evaluation of the catalytic concentrations of alanine aminotransferase (ALT), aspartate aminotransferase (AST), and alkaline phosphatase (ALP) was conducted in whole heparinized blood using the IDEXX VetTest analyser, as per the methodology established for this study (Wang et al., 2020). The determination of Ca^2+^ and Mg^2+^ concentrations were also carried out in whole heparinized blood with the IDEXX VetTest analyser. Inorganic phosphorus (P) content was determined in blood serum using the spectrophotometric method.

For Cu, Mn, Zn, Ca, Mg, and K analysis, 100 µL of the sample was decomposed in a microwave oven (Ethos ONE, Milestone, Italy) using 5 mL of 65% HNO_3_ suprapure (Merck) and 5 mL of water (1:1) at 210°C and 1,000 W for 30 min. An electrothermal atomic-absorption spectrometer (280Z AA, Agilent Technologies, Santa Clara, CA, United States, equipped with Zeeman correction) was used for the determination of Cu (324.7 nm) and Mn (279.5 nm), under the conditions recommended by the manufacturer. A 1% Pd/Mg (NO_3_)_2_ mixture was used as a modifier. For the determination of Zn (213.8 nm), Ca (422.7 nm), Mg (285.2 nm), and K (766.5 nm), a flame atomic-absorption spectrometer (240 FS AA, Agilent Technologies, Santa Clara, CA, United States) was employed. Acetylene-air flame was used for the determination of analytes, and 1% La_2_O_3_ was used as a modifier for Ca and Mg determination. The limits of detection (LODs) for the methods were 4.5 μg/L for Cu, 8.1 μg/L for Mn, 3.7 μg/L for Zn, 4.2 μg/L for Ca, 3.8 μg/L for Mg, and 2.5 μg/L for K. The reference material NIST 2670 was used for method validation.

### 2.6 Histopathological analysis

Due to ethical considerations and the nature of the study design, the calves were not sacrificed; therefore, no histopathological examination of liver or other organs was performed.

### 2.7 Fecal sample collection and analysis

Fecal samples were collected from all animals on day 0, i.e., prior to any exposure to the test article (including the habituation period), for the analysis of potential mycotoxin metabolites. Mycotoxin analysis (DON and ZEN) was performed prior to the commencement of the experiment, and a subsequent fecal sample collection was conducted on the 20th day of the experiment. The presence of mycotoxins in the feces was determined using an ELISA kit.

### 2.8 Statistics

The data were analysed using the statistical program R (R Core Team, 2022), and results are presented as the mean ± standard error of the mean. Differences between the means of the observed parameters at the beginning and at the end of the experiment were assessed using a paired t-test. Blood sample values from the experimental (T) and control (C) groups were analysed in this manner. The null hypothesis was rejected at p < 0.05.

## 3 Results

### 3.1 Feed efficiency during the experiment

The average weight, feed conversion ratio, and average daily weight gain were compared to evaluate feed efficiency and the effects of GO. All results related to calf growth parameters are presented in Table 4. At the beginning of the experiment, a reduction in feed intake of approximately 20% was observed in the experimental group (T). However, feed intake gradually increased over time and by the end of the study was comparable to that of the control group. The feeding efficiency of the calves in the experimental group varied: some animals accepted the GO-supplemented ration readily, while others showed lower feed acceptance. In contrast, only minor differences in feeding behavior were noted in the control group.

**TABLE 4:** Growth performance of calves (kg).

Time period	C	T
0 days	100.00 ± 1.30	102.00 ± 1.57
20 days	118.20 ± 2.41	115.40 ± 2.54
Weight gain		
Total	16.20 ± 0.32	15.40 ± 0.26
Day	0.81 ± 0.02	0.77 ± 0.02

The observed reduction in feed intake in the experimental group appeared to correspond with a lower average weight gain compared to the control group. However, it is important to note that the calves were weighed collectively as a group using a large-scale platform, and the mean weight per calf was calculated. As a result, individual variability in feed intake and weight gain could not be assessed, and the presented weight data should be interpreted with caution. Weighing was performed on Day 0 (the first day of the experimental feeding period) and again on Day 21 (the day after the experiment ended).

### 3.2 DON and ZEN levels in feces

The average levels of DON and ZEN were evaluated in feces and no significant differences were observed between C and T groups (Table 5).

**TABLE 5:** Content of mycotoxins in the feces of calves at the end of the experiment (mg/kg).

Mycotoxins	C	T
DON	0.274	<0.281
ZEN	0.05	<0.05

### 3.3 Biochemical parameters

To assess the health status and liver function, parameters were selected and compared between the C and T groups, as well as within each group (Table 6). No significant differences were observed between the groups, and the albumin content found corresponded to the reference values for cattle (Zaitsev et al., 2020; Carrillo-Muro et al., 2024; Amrollahi-Sharifabadi et al., 2018). ALT values were comparable in both groups. However, AST and ALP values were higher in the experimental group, although they remained within the reference range for healthy animals. In the T group, significant effects were observed for all the monitored liver enzyme parameters.

**TABLE 6:** Liver and antioxidant parameters.

Trait	Mean		p-value	Mean		p-value
	0 days	20 days		0 days	20 days	
		C			T	
Albumin (g/L)	37.90 ± 1.91	32.88 ± 1.05	0.0923	37.16 ± 3.41	33.74 ± 33.74	0.4410
ALT (µkat/L	1.33 ± 0.05	1.34 ± 0.03	0.6817	1.9 ± 0.02	1.33 ± 0.03	0.0220
AST (µkat/L)	1.22 ± 0.03	1.24 ± 0.06	0.7402	1.22 ± 0.02	1.35 ± 0.03	0.0273
ALP (µkat/L)	1.03 ± 0.05	1.05 ± 0.10	0.8630	1.05 ± 0.06	1.38 ± 0.02	0.0154
GPx (µkat/L)	820.60 ± 24.03	779.20 ± 61.53	0.5249	712.80 ± 46.70	591.80 ± 66.96	0.0113
SOD (U/mL)	1748.40 ± 88.05	1,679.00 ± 145.27	0.6720	1,741.60 ± 90.78	1,717.40 ± 111.42	0.8982

The catalytic concentrations of glutathione peroxidase (GPx) were assessed spectrophotometrically. No significant differences were observed for the antioxidant enzyme GPx, although the GO-supplemented diet led to a significant reduction in GPx levels. Superoxide dismutase (SOD) catalytic concentrations were also evaluated. While SOD concentrations were higher in the experimental group, this finding was not statistically significant. The SOD levels were consistent with reference values for healthy animals.

The mineral parameters in both the experimental and control groups are shown in Table 7. No significant differences were observed between the C and T groups for Ca and P, and the measured levels of microelements were consistent with the physiological values for cattle. No significant differences were found in the determination of K, Na, and Cl. The levels of these micronutrients were within the bovine reference values. Similarly, no significant differences were observed for Fe, Cu, and Zn, and the measured content of these elements corresponded to the reference values for cattle.

**TABLE 7:** Analysis of mineral substances.

Trait	Mean		p-value	Mean		p-value
	0 days	20 days		0 days	20 days	
		C			T	
Ca (mmol/L)	2.44 ± 0.12	2.50 ± 0.09	0.6263	2.94 ± 0.34	2.39 ± 0.06	0.1798
P (mmol/L)	1.77 ± 0.05	1.67 ± 0.08	0.3157	1.76 ± 0.08	1.60 ± 0.10	0.1811
Mg (mmol/L)	0.62 ± 0.06	0.81 ± 0.04	0.02851	0.65 ± 0.05	0.70 ± 0.03	0.3899
K (mmol/L)	5.00 ± 0.22	5.32 ± 0.21	0.3625	5.12 ± 0.16	5.81 ± 0.52	0.2085
Na (mmol/L)	144.98 ± 1.57	143.78 ± 5.22	0.7879	151.93 ± 1.59	149.62 ± 2.79	0.5577
Cl (mmol/L)	82.82 ± 1.46	86.04 ± 2.21	0.1203	79.62 ± 1.42	87.04 ± 3.64	0.1085
Fe (mmol/L)	30.82 ± 0.72	28.46 ± 2.29	0.2952	35.48 ± 0.28	29.68 ± 1.83	0.04086
Cu (mmol/L)	13.35 ± 0.29	13.58 ± 0.25	0.6224	14.96 ± 0.61	13.94 ± 0.20	0.08439
Zn (mmol/L)	14.60 ± 0.26	14.32 ± 0.22	0.2212	15.80 ± 0.11	13.76 ± 0.24	0.000562

## 4 Discussion

Graphene-based materials, such as graphene oxide (GO), are known for their excellent adsorption properties toward both organic and inorganic substances. However, their non-specific adsorption capacity can adsorb essential micronutrients, potentially resulting in nutrient depletion and affecting the nutritional status of organisms (Bytešníková et al., 2023; Long et al., 2012). The underlying mechanism involves the interaction of GO’s reactive oxygen-containing functional groups with small molecules, facilitating both covalent and non-covalent binding. Such interactions are not highly selective, thereby affecting a wide range of solutes, including essential nutrients (Amrollahi-Sharifabadi et al., 2018). GO has a special chemical modification with highly reactive oxygen functional groups, effectively acting as a stabilizing agent in water. This modification allows GO to covalently bind to small molecules. Physical adsorption, however, is only weakly specific and represents a general phenomenon affecting a wide range of small molecule solutes, including amino acids (AAs) and vitamins. The extent of adsorption is most pronounced for solutes present at low initial concentrations and with molecular structures favouring interactions with graphitic carbon. Key factors influencing adsorption include hydrophobicity, molecular planarity/sp^2^ hybridization facilitating π-π interactions, and a positive charge, which is opposite to the typically negative charge of carbon surfaces at neutral pH (Guo et al., 2008). Long et al. (2012) investigated the mechanisms of carbon nanotube (CNT) toxicity to algae. They found that nutrient concentrations were reduced in media exposed to CNTs compared to the control, potentially affecting nutrient availability and algal growth. This suggests that CNTs–and potentially other nanomaterials such as graphene oxide (GO) – may interfere with nutrient absorption and metabolism (Long et al., 2012). In our study, the experimental group (T), which received GO-supplemented feed, achieved a total weight gain of 15.40 kg, while the control group (C) gained 16.20 kg over the same period. When expressed as average daily gain (ADG) per calf, group T gained 0.77 kg/day, compared to 0.81 kg/day in group C. Although both groups were initially comparable and group T underwent a 3-day adaptation period to the GO-supplemented diet, a reduction in ADG of 0.26 kg was observed in the experimental group. Taking the control group’s gain as 100%, this corresponds to a 29.9% reduction in weight gain, which may indicate that GO negatively affected nutrient utilization and growth performance. This decrease may be partially explained by the observed reductions in plasma levels of essential micronutrients, particularly zinc and iron, which are both known to play key roles in growth, immune function, and metabolic activity in calves (Budny-Walczak et al., 2023; Chang et al., 2020; Wo et al., 2022; Rajaei-Sharifabadi et al., 2024). Similar research on nutrient depletion was conducted by Zhao et al. (2017a), who reported that the indirect toxicity of graphene particles is often driven by their ability to adsorb nutrients from the environment. They emphasized nutrient depletion as a critical factor in assessing the negative impact of graphene particles, noting reduced cell growth in media exposed to graphene due to reduced nutrient availability (Zhao et al., 2017a). This finding is consistent with the research by Amrollahi-Sharifabadi et al. (2018), who observed changes in body weight gain in rats following intraperitoneal exposure to GO over a 21-day period (Amrollahi-Sharifabadi et al., 2018). Fu et al. (2015) observed a reduction in body weight in mice orally administered GO. They attributed this effect to GO-induced dysfunction in the intestinal tract, as well as abnormal blood biochemistry. Their results suggest potential toxicity associated with oral administration of GO (Fu et al., 2015). While the study by Amrollahi-Sharifabadi et al. (2018) involved intraperitoneal exposure, the research by Fu et al. (2015) used oral administration, which more closely mimics the exposure route in our study (Rhazouani et al., 2021).However, this finding contrasts with the research by Yang et al. (2010), Yang et al. (2013), Yang et al. (2011), who found no significant decrease in body weight in mice after 90 days of exposure to PEG-graphene nanolayers. In their studies, PEGylated graphene oxide was primarily administered intravenously, which differs from the oral route used in our experiment and may explain the discrepancy in observed weight-related outcomes (Yang et al., 2010; Yang et al., 2011; Yang et al., 2013).

There is limited information in the literature regarding the adsorption properties of minerals on GO and its impact on mineral metabolism and the health status of ruminants. Some studies indicate that mineral adsorption can reach up to 70% (Horky et al., 2020; Zhao et al., 2017b) which can be attributed to the functional groups on its surface, which are suitable for interaction with both cations and anions (Kanayama et al., 2014). In another study by Yang et al. (2016), they found that epoxy groups trap metal cations and that these metal cations can easily interact with the aromatic rings on GO via cation-π bonding. The metal cations can bind to the hydrophobic aromatic surface through cation-π interactions and electrostatic attraction (Yang et al., 2016). According to research by Liu et al. (2019), which focused on the interaction between GO and minerals, their study concluded that pH, ionic strength, and temperature significantly affect the adsorption behaviour of GO. They attributed the interactions of GO with minerals to electrostatic interactions and hydrogen bonding. Their results also showed that the interaction between GO and minerals was irreversible. Based on these findings, both temperature and ionic strength significantly influence adsorption and desorption (Liu et al., 2019). Zhao et al. (2017b) found that the concentrations of Fe, Cu, and Zn remained unchanged for microelements, while the concentrations of Mg^2+^ and Ca^2+^ were significantly reduced. They explained this by the fact that Fe, Cu, and Zn have higher electronegativity than Mg^2+^ and Ca^2+^ (Zhao et al., 2017a). This does not entirely agree with the research of Zhao et al. (2017a), who focused on the feeding of montmorillonite in pig diets and its effect on the organism. They found that supplementation significantly reduced the levels of Mg, Zn, and Cu in the serum of pigs (Zhao et al., 2017b). Among the essential minerals, *in vitro* research by Skaličková et al. (2020) demonstrated that GO dominantly adsorbs Mn (90%), Cu (80%), Zn (60%), Ca (40%), K (35%), and Mg (30%) (Horky et al., 2020), making these elements particularly susceptible to depletion (Horky et al., 2020). Amrollahi-Sharifabadi et al. (2018) observed reduced Na^+^ and Ca^2+^ levels in their study (Amrollahi-Sharifabadi et al., 2018). High Ca^2+^ affinity is also demonstrated in the study by Kanayama et al. (2014). Folic acid, pyridoxine, and niacinamide readily adsorb to graphene, with folic acid adsorbing even at very low graphene doses (<10 μg/mL). These micronutrients contain planar conjugated units that promote adsorption through hydrophobic and π-π interactions (Yang and Xing, 2010; Creighton et al., 2013). Since these results are generated *in vitro* or modelled *in vivo* in the gastrointestinal tract (GIT) of pigs, rats, or mice, they cannot be fully compared with *in vivo* results in calves. Graphene oxide (GO) is a highly effective adsorbent with a strong affinity for various feed components, including micro- and macronutrients. Studies have demonstrated that GO can adsorb significant proportions of essential minerals such as manganese, copper, zinc, calcium, potassium, and magnesium (Horky et al., 2020; Zhang et al., 2016). Moreover, GO interacts with vitamins containing aromatic ring structures through π–π interactions and hydrophobic forces (Creighton et al., 2013), as well as with positively charged amino acids via electrostatic and hydrogen bonding (Nassef et al., 2018; Mantovani et al., 2022). These cross-reactivities may influence the bioavailability and metabolism of these nutrients, warranting thorough investigation into their implications for animal nutrition.

Some authors have raised concerns that GO may induce oxidative stress in animal cells, which could represent another mechanism of GO cytotoxicity. GPx is a family of enzymes that scavenge or neutralize H_2_O_2_, with the endogenous GPx enzyme being the primary antioxidant. GPx levels depend on reduced GSH to perform its function, providing reducing equivalents, and thus catalysing the conversion of H_2_O_2_ to H_2_O. Simultaneously, higher GPx activity can affect GSH levels (Mohideen et al., 2023). Research by Zhao et al. (2017b) examined the effect of montmorillonite on the health status of pigs and observed a decrease in antioxidant and GPx activity, while malondialdehyde (MDA) and superoxide dismutase (SOD) activity significantly increased (Zhao et al., 2017b). Superoxide dismutase (SOD) is a class of enzymes that maintain a dynamic balance between the production and elimination of biological oxidants in the body, thereby preventing the toxic effects of free radicals (Zheng et al., 2023). In our investigation, no significant changes in SOD levels were observed in either group. Our results contrast with the findings of Patlolla et al. (2016) who reported increased SOD levels in rats treated with GO. Their study also showed elevated levels of GPx and catalase (CAT), suggesting an adaptive mechanism to mitigate the toxic effects of H_2_O_2_. The activities of CAT, SOD, and GPx were found to increase in a dose-dependent manner compared to the control group (Patlolla et al., 2016). GPx plays a critical role in the detoxification of lower concentrations of H_2_O_2_, whereas catalase (CAT) becomes active when the GPx pathway is saturated with the substrate or when H_2_O_2_ is present in excess (Mohideen et al., 2023). In our study, we observed a significant change in GPx levels, with a decrease in enzyme activity in the supplemented calves, while no change in SOD levels was detected. GPx levels in calves remained within the reference values for cattle (Yu et al., 2019). However, we acknowledge that the assessment of oxidative stress in this study was limited to GPx and SOD. Additional antioxidant markers such as catalase (CAT), malondialdehyde (MDA), or total antioxidant capacity (TAC) were not included due to technical constraints. This limits the depth of interpretation, and future studies should incorporate a broader oxidative stress panel to provide a more comprehensive evaluation of GO-related redox effects.

Previous studies have shown that the main target organs of nanoparticles in mice, when administered orally, are the liver, spleen, kidneys, and lungs. The distribution depends on factors such as the route of administration, dose, and other variables (Ou et al., 2016; Liao et al., 2018; Jasim et al., 2016; Liu et al., 2012; Wang et al., 2011; Yang et al., 2010; Yang et al., 2011; Yang et al., 2013; Wen et al., 2015; Aguado-Henche et al., 2022; Yang et al., 2013; Li et al., 2018). Research by Zhang et al. (2011) does not support the deposition of GO in the liver and spleen. According to their findings, GO was deposited in the lungs (Zhang et al., 2011). In a study by Amrollahi-Sharifabadi et al. (2018), in which graphene oxide was administered intraperitoneally at an extremely high dose (500 mg/kg), histopathological changes were observed in the liver, kidney, lung, and small intestine. Elevated levels of ALT, AST, and ALP were also noted (Amrollahi-Sharifabadi et al., 2018). In a study by Aguado-Henche et al. (2022), an increase in AST and albumin levels was observed in a group administered intraperitoneally with GO for 30 days (Aguado-Henche et al., 2022). Significant increases in serum AST, ALT, and ALP levels were observed in Wistar rats exposed to graphene oxide, as reported by Nirmal et al. (2021). In the study by Fu et al. (2015) no statistically significant differences were observed in ALT and AST levels between the control group and the group treated with GO at 0.05 mg/mL. However, higher AST levels were observed in mice treated with GO at 0.5 mg/mL, although the difference was not statistically significant (Fu et al., 2015). Research by Yang et al. (2010), Yang et al. (2013), Yang et al. (2011) showed similar results, with no significant changes observed in blood biochemistry or hematology. Additionally, liver function markers indicated no apparent hepatic toxicity (Yang et al., 2010; Yang et al., 2013; Yang et al., 2011). In a study by Yang et al. (2013), mice administered GO intravenously showed a slight decrease in ALT levels, which fell slightly below the reference values. However, these differences were not physiologically significant when compared to untreated controls. Other liver enzymes, such as ALP and AST, showed no statistically significant changes (Yang et al., 2013). No significant differences in AST levels were observed, even in the study by Horký et al. (2021). Liver enzyme levels in calves are expected to range from ALT (5.2–15.3 U/L), and in adult cattle, from ALT (11–40 U/L) and AST (19.0–63.3 U/L) to AST (78–132 U/L) (Yu et al., 2019). In our investigation, significant changes were observed in liver enzymes, with a decrease in ALT, an increase in AST, and an increase in ALP, all remaining within the reference range for cattle. Elevated levels of ALT, AST, and ALP are well known to be associated with inflammation and liver tissue damage (Gowda et al., 2009; Giannini et al., 2005). Increased levels of albumin and AST can indicate liver damage; however, in our case, the levels remained within the reference range. Several authors agree that graphene and GO can induce liver damage (Yang et al., 2013; Li et al., 2016). Our study confirmed some effects of GO on the liver. During the study, it was observed that general health parameters were like those of the control group, suggesting that GO does not induce drastic toxicity to overall body metabolism. However, serum levels of liver function enzymes were elevated, which could indicate inflammation or liver damage. Although these changes were statistically significant, all values remained within the physiological reference range. Therefore, the observed effects may reflect a mild biological response rather than clinically relevant hepatic pathology. We acknowledge that the study did not include a washout period, which could have provided further insights into the reversibility of these effects. Therefore, we recommend that future studies incorporate a post-treatment monitoring phase to determine whether the elevated enzyme levels return to baseline, helping to distinguish between transient physiological responses and direct toxic effects of GO. Another limitation of this study is the absence of histopathological data to corroborate the biochemical indications of potential hepatic stress observed through elevated liver enzyme levels. Since the calves were not euthanized, tissue-level analyses were not feasible. This limits the ability to fully assess possible morphologic organ damage and should be addressed in future research.

As demonstrated by other studies in mammalian models, there is potential toxicity to various organs in the body. To clarify the actual organ toxicity and overall toxicity to the organism, additional factors related to local toxicity must be considered. Furthermore, the analysis of more sensitive parameters, such as molecular effects or the impact on the microbiota in the rumen or gut, is essential (Ou et al., 2016; Jia et al., 2019; Fu et al., 2015). Furthermore, the study did not include a full physicochemical characterization of the graphene oxide used. Although basic information was provided by the supplier, additional parameters such as zeta potential, oxidation degree, and purity were not analyzed. This limits reproducibility and mechanistic interpretation. Future studies should incorporate a more detailed characterization of GO materials. These studies do not fully align with our results, which may be attributed to various factors and the intrinsic physicochemical properties of GO, such as surface functional groups, charge, size, and structural defects, as well as differences in its size, functionalization, and purification. These factors affect its behaviour both *in vitro* and *in vivo* and influence its toxicity to biological systems (Dreyer et al., 2010; Liu et al., 2008; Rhazouani et al., 2021).

Finally, the small sample size used in this pilot study significantly limits the statistical power and generalizability of the findings. While the observed effects provide valuable preliminary insights, they must be interpreted with caution. Larger-scale studies will be necessary to confirm these results and to evaluate the reproducibility and broader biological relevance of GO supplementation in livestock. In addition, the short duration of the study (20 days) limits conclusions about potential chronic or long-term effects of GO supplementation. Future experiments should include extended observation periods to better assess cumulative toxicity and delayed biological responses.

Another important limitation of this study is the relatively high dose of graphene oxide (30 g per calf per day, corresponding to approximately 30 mg/kg of feed), which may not reflect realistic concentrations used in practical feed applications. This dosage was deliberately selected based on previously published *in vitro* and *in vivo* studies (Horky et al., 2020; Shen et al., 2022; Liang et al., 2015) in animal models, with the aim of inducing detectable biological responses in the context of a pilot trial. Due to financial and logistical constraints, only a single dose level was evaluated. This experimental design limits our ability to assess dose-dependent effects or define a potential safety threshold. Future studies should therefore include a wider range of doses, particularly lower and more field-relevant concentrations, to better evaluate the efficacy and safety profile of GO under real-world livestock feeding conditions. Moreover, this study did not include long-term monitoring of mineral homeostasis, immune response, or fecal microbiota composition, which could provide further insight into the chronic effects and systemic impact of GO supplementation. Future research should incorporate extended follow-up and additional biomarkers to capture these important aspects of animal health. Moreover, the study did not directly assess the mycotoxin-binding efficacy of graphene oxide *in vivo*, as the naturally occurring mycotoxin levels in the feed were low and did not allow for meaningful evaluation. This represents a limitation in verifying one of the proposed functional properties of GO. Future studies should include feed with defined levels of mycotoxin contamination to properly assess the adsorption capacity and potential protective effects of GO under realistic conditions.

## 5 Conclusion

So far, no systematic studies on this subject have been published. According to a review of various papers, this study is the first to focus on the administration of GO in feed as an adsorbent of mycotoxins in calves and its effects on their general health. Feed and food contamination is—and will continue to be—a worldwide problem, and feedstuffs create complex matrices that can complicate the adsorption of mycotoxins on GO. Both macro- and micronutrients in the feed can bind to GO, potentially hindering mycotoxin adsorption.

In our study, we observed changes in the growth performance of calves exposed to GO, which may indicate a limitation of GIT nutrient access. However, within the biochemical parameters, we did not observe any statistically significant effects of GO on the health status of the calves. Slight, non-significant upward trends in liver enzymes (ALP, ALT, AST) were noted in the experimental group, while the GPx value showed significant reduction. The adsorption capacity of GO can be influenced by the presence of a biological matrix, from its mixing into the animal’s ration to its passage across the GIT in cattle. Furthermore, GO exhibits a non-specific effect on micronutrients, particularly Zn and Fe. A decrease in Zn and Fe concentrations was observed in the experimental group, which may indicate a potential effect of graphene oxide (GO) on micronutrient levels.

Considering the observed mild changes in liver enzymes and the absence of significant signs of toxicity at the overall health level, it can be assumed that a single exposure to GO at this dose does not cause major metabolic disruption. However, since only one dose was tested, we acknowledge this as a limitation of the study. Additionally, the absence of a follow-up period after the withdrawal of GO from the feed represents another limitation, as it prevents the assessment of potential reversibility of observed effects. We recommend that future research include multiple dosage levels, ideally including lower doses, and consider incorporating a recovery phase to better determine the threshold of toxicity, dose-dependent effects of GO, and the possible reversibility of its impact.

Additionally, *in vivo* conditions, such as GO dose, exposure time, cell or animal type, and the technique used to assess cell viability, may influence the biocompatibility and toxicity of GO. GO has an excellent adsorption capacity, and by modifying or functionalizing its groups, it could be a promising option for the adsorption of mycotoxins in an organism. When comparing with other *in vivo* results, mostly focused on rats or piglets, it is important to note that our research was focused on ruminants, which have a different digestion mechanism. Therefore, there is room for further research in this area.

## Data Availability

The original contributions presented in the study are included in the article/Supplementary Material, further inquiries can be directed to the corresponding author.
